# AI in pharmacy education: a comparative visualization analysis of global and Chinese research trends

**DOI:** 10.3389/fmed.2026.1866054

**Published:** 2026-06-30

**Authors:** Meng You, Chunmeng Sun, Wei Hu

**Affiliations:** 1 School of Marxism, China Pharmaceutical University, Nanjing, China; 2 Department of Pharmaceutics, School of Pharmacy, China Pharmaceutical University, Nanjing, China; 3 Institute of Biomedical and Pharmaceutical Strategy Research, China Pharmaceutical University, Nanjing, China

**Keywords:** artificial intelligence, competency-based education, curriculum reform, digital literacy, pharmacy education, visualization analysis

## Abstract

**Background:**

The rapid integration of Artificial Intelligence (AI) into healthcare necessitates a paradigm shift in pharmacy education to prepare future-ready professionals. However, the global trajectory of this educational evolution and the distinct strategic approaches between different regions remain under-explored. This study aims to systematically map the global and Chinese research landscapes, identifying evolutionary trends, pedagogical shifts, and core competency requirements in the intelligence era.

**Methods:**

A comparative visualization analysis was conducted using CiteSpace (6.4.R1) on literature retrieved from the Web of Science (WoS) Core Collection and China National Knowledge Infrastructure (CNKI) covering publications up to December 31, 2025 (data retrieval was conducted in early 2026). We employed co-occurrence clustering and burst detection to analyze publication trends, institutional collaborations, and keyword evolution. The study specifically examined the divergence and convergence of educational strategies between global and Chinese contexts.

**Results:**

A total of 240 English and 118 Chinese high-relevance articles were analyzed, revealing a rapid growth trajectory characterized by a logarithmic pattern, with universities as primary research hubs. The analysis identified three critical pedagogical transitions: Assessment Transformation: A shift from rote memorization to complex clinical reasoning, driven by Gen AI tools; Pedagogical Innovation: The integration of “Virtual Reality” and “Immersive Teaching,” signaling a move toward technology-enhanced experiential learning; and Competency Redefinition: A pivot from traditional pharmacology knowledge to “Digital Literacy” and “Interdisciplinary Abilities.” Notably, comparative analysis revealed that while global research focuses heavily on the technical integration of AI in clinical practice, Chinese literature demonstrates a stronger orientation toward policy-driven curriculum reform and top-level design.

**Conclusion:**

AI is increasingly recognized as a key catalyst associated with pedagogical evolution rather than merely a technological adjunct. Notably, while global efforts prioritize the technical integration of AI into clinical tools, Chinese initiatives emphasize top-level policy-driven curriculum reform. To bridge the gap between education and practice, institutions must transition from tool-based instruction to a deep integration of AI ethics, data logic, and clinical decision-making. This study provides an evidence-based roadmap for educators to align curricula with the accelerating digital transformation of the pharmacy profession.

## Introduction

With the improvement of computing power and the accumulation of massive pharmaceutical data, Artificial Intelligence (AI) has penetrated every aspect of drug development, clinical medication guidance, and pharmaceutical education. In the field of drug discovery and clinical pharmacy, AI has significantly shortened the screening cycle of lead compounds (1, 2) and transformed traditional prescription review models (3, 4). However, as the paradigm of the pharmacy profession shifts towards digital and intelligent practices, the pedagogical approaches in pharmacy education must evolve accordingly. Despite the increasing integration of AI into educational settings, research specifically focusing on the intersection of AI and pharmacy education remains underexplored.

To establish the current landscape of this field, previous bibliometric studies have mapped the application of AI in general medical education or focused broadly on health professions (5, 6). Some existing reviews have analyzed pharmaceutical AI trends relying solely on a single database (7). A thorough literature review reveals a significant research gap: there is currently no prior bibliometric study that explicitly compares the evolutionary trajectories, focal points, and collaborative networks of AI in pharmacy education between the global context and the Chinese context.

Addressing this gap is crucial because the following challenges and disparities remain in current research: the imbalance between the breadth and depth of research, where existing literature often focuses on the application of specific algorithms rather than macro-level knowledge graph analysis (8, 9); the divergence in research hotspots, where international research (Web of Science, WoS) tends to focus on cutting-edge technologies (e.g., generative AI in molecular design education), while domestic research (China CNKI) emphasizes policy-driven smart hospital construction and pharmaceutical talent cultivation reforms (10, 11); the limitations of cross-institutional collaboration, exhibiting low inter-institutional collaboration density and predominantly independent research (12, 13). Therefore, drawing on visualization analysis methods to systematically conduct a comparative review (WoS vs. CNKI) of AI in pharmacy education holds significant academic value for grasping disciplinary frontiers and predicting future research trends.

To systematically guide this mapping review, the research question was formulated by adapting the PICOS framework for a bibliometric analysis (14, 15): Population (P): Published literature and research metadata in the field of pharmacy and pharmacy education; Intervention (I): Application and integration of AI technologies; Comparator (C): Educational strategies, policies, and research trends between the global context (WoS) and the Chinese context (CNKI); Outcomes (O): Publication growth trends, institutional collaborative networks, pedagogical shifts, and keyword evolutionary bursts; Study Design (S): Bibliometric visualization and systematic mapping review.

## Methods

### Search strategy

To investigate the current advancements in AI applications within the Chinese and international pharmacy fields, a comprehensive literature search was conducted. The China National Knowledge Infrastructure (CNKI) database and the Web of Science (WoS) Core Collection were purposefully selected as the primary data sources. WoS was chosen for its rigorous indexing and comprehensive coverage of interdisciplinary research (16), which is particularly suited for capturing the educational and socio-technical dimensions of pharmacy AI. Similarly, CNKI is the most authoritative database for analyzing domestic educational reforms. The retrieval time frame for both databases was set from January 01, 2017, to December 31, 2025. For CNKI, the retrieval strategy focused on main topics: Themes = (artificial intelligence [人工智能]) AND (pharmacy [药学]). For the WoS Core Collection, the retrieval approach was TS = (“Artificial Intelligence”) AND TS = (“pharmacy”), with the search refinement language confined to English.

### Data screening and preprocessing

Clear inclusion and exclusion criteria were established to ensure high academic quality. The inclusion criteria for document types were exclusively limited to “Article” and “Review Article.” Other document types, such as editorial materials, meeting abstracts, news reports, notices to contributors, conferences, forums, and dissertations, were explicitly excluded. Following the removal of duplicate papers via CiteSpace software, a final group of 240 valid English papers and 118 Chinese papers was acquired.

During the data preprocessing phase, author names, country names, and affiliations were standardized to resolve inconsistencies (e.g., correcting typographical errors and unifying initials with full names). Keywords were refined by eliminating duplicates and categorizing strictly synonymous terms under uniform terminology (for instance, standardizing abbreviations like “AI” to “artificial intelligence,” and unifying variations in institutional naming into their primary university affiliations) to ensure analytical credibility and robust thesaurus construction (17). It is important to note that specific AI applications and subtypes (e.g., “ChatGPT”) were retained as distinct keyword nodes to preserve data granularity, recognizing that specific models are subtypes rather than exact synonyms of broader categories like “generative AI.” Furthermore, since “pharmacy” and “artificial intelligence” acted as broad search strategy terms, they were explicitly excluded from the high-frequency keyword analysis and clustering processes to avoid analytical redundancy and conceptual confusion.

### Keyword analysis method

- Keywords co-occurrence analysis method. In the analysis of keyword co-occurrence keywords were combined according to their relevance to the research content. It should be noted that high-frequency keywords do not always accurately identify research hotspots. In the keyword co-occurrence analysis nodes with high centrality are marked by purple rings and the thickness of these rings indicates the centrality value. This visual method emphasizes possible cross-disciplinary connections and emerging links within the research field
- Keywords burst analysis method. The phrase “keyword bursts” denotes a substantial increase in how often keywords appear allowing for the detection of leading-edge content in the research domain (18). In this study we carried out an analysis of keyword bursts to pinpoint the top 25 keywords in both Chinese and English publications which can be grouped into various categories according to their content
- Keywords clustering and time diagram. The CiteSpace software was used to cluster keywords and Panthfinder and Pruning were applied to the merged network to generate keyword clustering network diagram and keyword time diagram and the top 50 keywords by annual frequency were employed for constructing the keyword clustering networks. The keyword clustering can be categorized into different groups based on their content

### Data analysis and visualization procedures

We utilized CiteSpace (6.4.R1) as the primary analytical tool to construct and visualize bibliometric networks. The time partition length (Slice Length) was configured to 1 year. Node types including author, institution, and keyword were selected for respective analyses. To balance network clarity and data retention, the selection criteria were defined using a modified g-index (k = 25), and pathfinder network scaling along with pruning sliced networks were applied to reduce structural complexity and clarify the visual networks.

Criteria for visualization and interpretation:

- Institutional and Author Co-occurrence: Node size indicates publication volume, while connecting lines reflect the intensity of collaboration. We applied Price’s law to define high-producing authors (individuals with≥2 publications in this dataset);
- Keyword Co-occurrence and Clustering: Nodes with high betweenness centrality (≥ 0.1) are marked by purple rings signifying their role as critical hubs connecting different research themes. Keyword clustering was employed to generate network and timeline diagrams;
- Keyword Burst Analysis: The burst detection algorithm was applied to pinpoint leading-edge content. We selected the top 25 keywords with the strongest citation bursts. This specific threshold (Top 25) was justified based on the frequency distribution of our dataset and precedents in prior bibliometric studies as it optimally balances network stability with visual interpretability preventing clutter while effectively highlighting the most significant thematic shifts.

Our overall approach is situated within established research practices in the library and information science (LIS) domain (19, 20).

## Results

### Study characteristics and PRISMA flow

**Figure 1:**
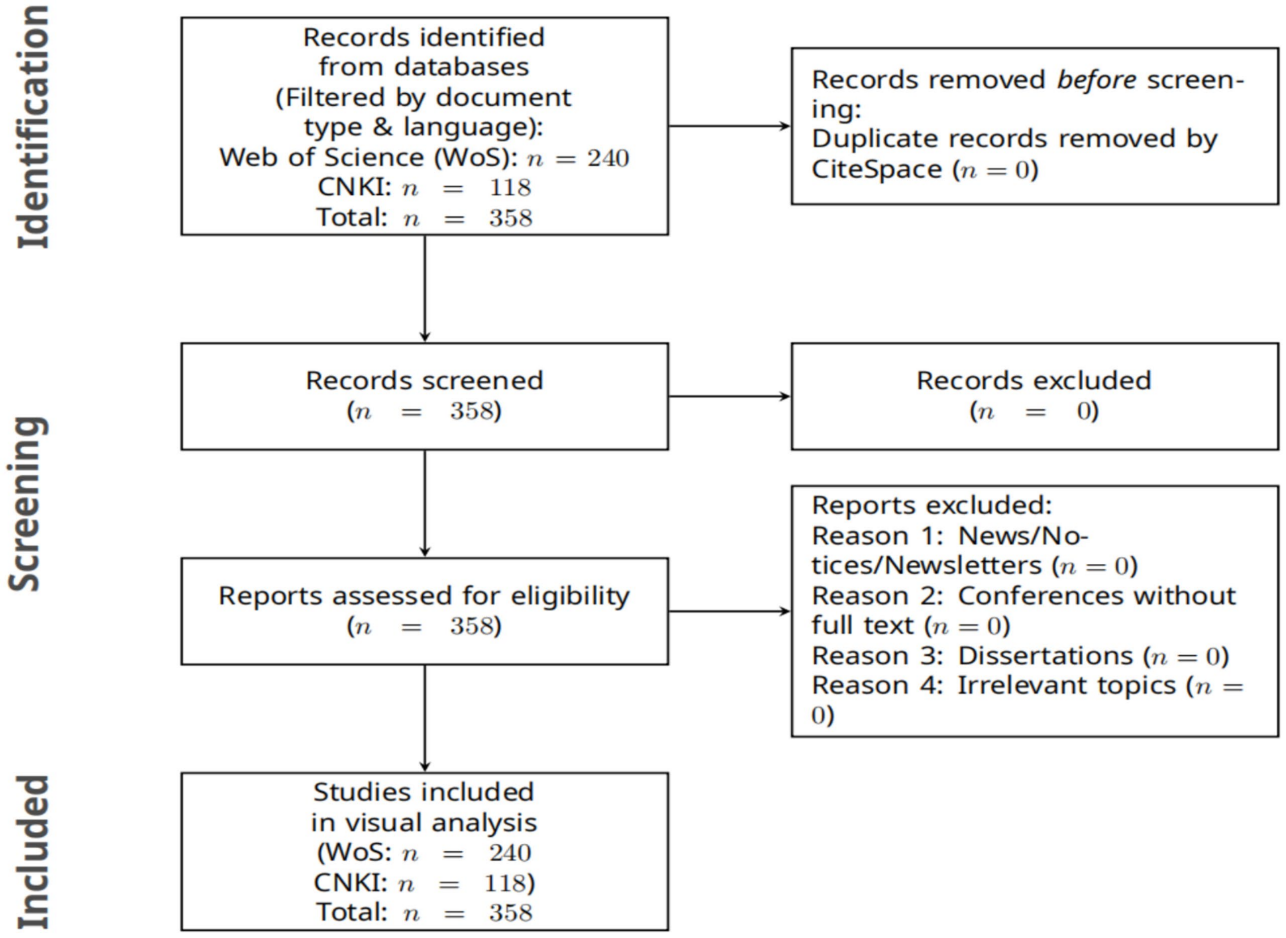
Literature retrieval strategy and screening flowchart based on the PRISMA 2020 statement.

Based on the defined search strategy and selection criteria, the initial search yielded a preliminary pool of literature. After removing duplicates and applying the exclusion criteria (e.g., excluding news reports, conferences, and irrelevant topics), a total of 240 valid English papers from WoS and 118 valid Chinese articles from CNKI were finalized for the visual analysis. The complete screening and selection process is documented in the PRISMA flow diagram (Figure 1). Furthermore, the automated deduplication and standardized data cleaning minimized the risk of bias within the included datasets, ensuring robust synthesized findings.

### Publication trend

**Figure 2:**
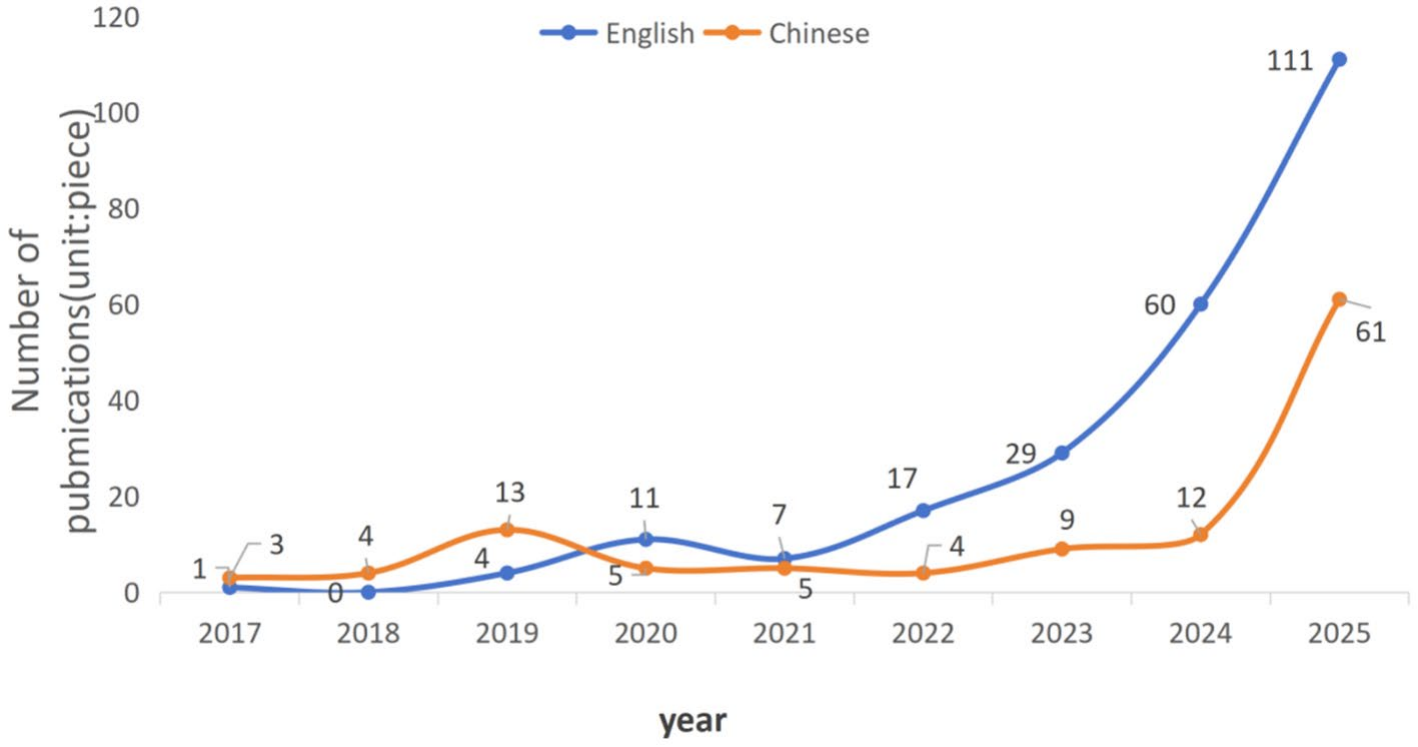
Annual number of published pharmacy literature related to AI technique. The solid lines denote the actual publication counts per year, with the numbers indicating the exact figures.

The number and trend of published papers in a specific field can serve as indicators of the developmental stage, research trends, and knowledge maturity within that field. Publication counts show an overall upward trend across both datasets, reflecting the growing scholarly interest in this domain. According to the trend chart (Figure 2), the application of Artificial Intelligence in Pharmacy has undergone stages of initial, steady, and rapid development.

### Authors

**Figure 3:**
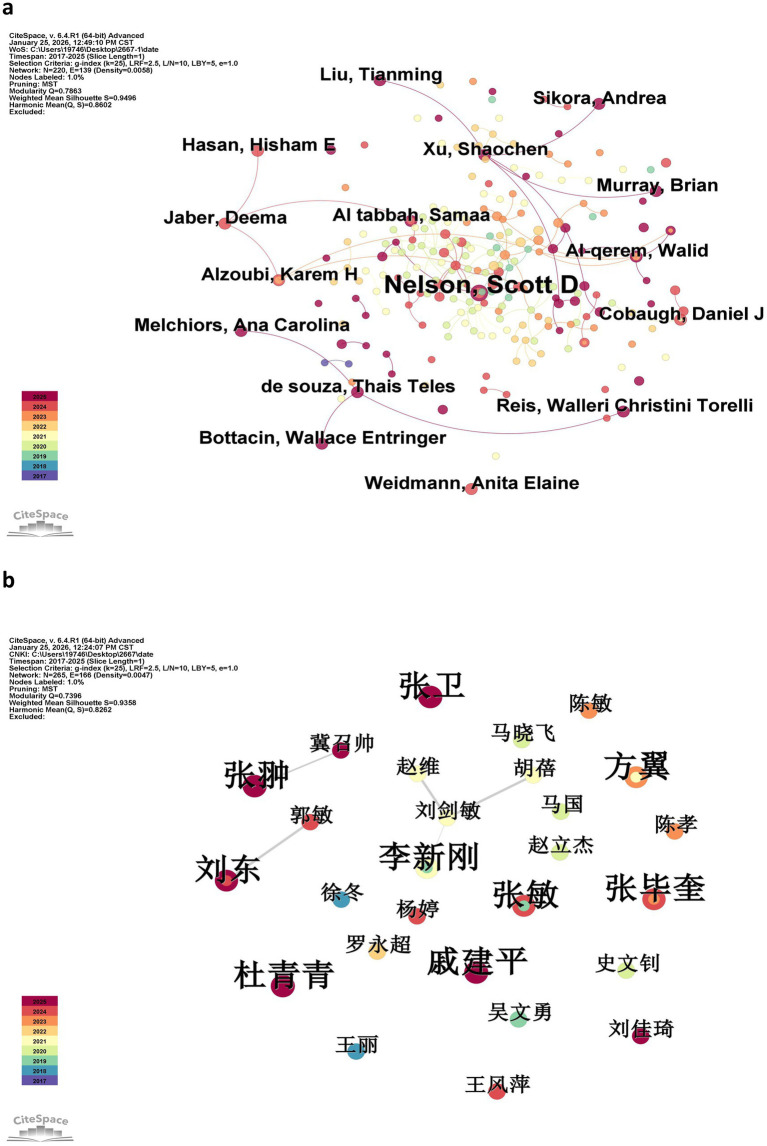
Atlas of English **(a)** and Chinese **(b)** co-authors in pharmacy related to artificial intelligence. In Chinese, the terms are organized as follows: “方翼” for “Yi Fang,” “李新刚”for Xingang Li (in Chinese), “张敏” for Min Zhang,” 张卫”for “Wei Zhang,” “张毕奎”for “Bikui Zhang” and “张翀”for “Chong Zhang,” “杜青青”for”Qingqing Du,” “刘东” for “Dong Liu,” “戚建平” for “Jianping Qi”.

The author knowledge map of Chinese and English literature was generated using CiteSpace software (Figure 3). The analysis revealed that the network consisted of 265 nodes (N), 566 connections (E), and a density of 0.0162 for Chinese literature, while for English literature, there were 220 nodes, 365 connections, and a density of 0.0152. Overall, collaboration between researchers in Chinese and English literature was limited, with most studies being conducted independently. However, based on Price’s law, we identified a notable subset of high-producing authors within the dataset, comprising nine in the Chinese literature and forty-nine in the English literature (Table 1 provides detailed information).

**Table 1:** Authors with more than or equal to 2 papers published in English and Chinese.

English					Chinese				
No	Count	Centrality	Year	Authors	No	count	Centrality	Year	Authors
1	6	0.01	2020	Nelson, Scott D	1	2	0	2019	张敏
2	3	0	2025	Murray, Brian	2	2	0	2025	张卫
3	3	0	2025	Reis, Walleri Christini Torelli	3	2	0	2023	张毕奎
4	3	0	2025	Xu, Shaochen	4	2	0	2025	张翀
5	3	0	2024	Hasan, Hisham E	5	2	0	2025	杜青青
6	3	0	2024	Al tabbah, Samaa	6	2	0	2024	刘东
7	3	0	2025	Liu, Tianming	7	2	0	2025	戚建平
8	3	0	2025	Sikora, Andrea	8	2	0	2021	方翼
9	3	0	2025	Bottacin, Wallace Entringer	9	2	0	2019	李新刚
10	3	0.01	2023	Al-qerem, Walid					
11	3	0	2025	Melchiors, Ana Carolina					
12	3	0	2025	de souza, Thais Teles					
13	3	0	2024	Weidmann, Anita Elaine					
14	3	0	2020	Cobaugh, Daniel J					
15	3	0.01	2023	Alzoubi, Karem H					
16	3	0	2024	Jaber, Deema					
17	2	0	2025	Mclaughlin, Jacqueline E					
18	2	0	2023	Al-ashwal, Fahmi Y					
19	2	0	2024	Kim, Eunyoung					
20	2	0	2022	Roosan, Moom R					
21	2	0	2024	Jin, Hye Kyung					
22	2	0	2024	Cunningham, Francesca E					
23	2	0	2025	Devraj, Radhika					
24	2	0	2025	Mattingly ii, T Joseph					
25	2	0	2025	Farland, Michelle Z					
26	2	0	2023	Jarab, Anan S					
27	2	0	2025	Li, Xiang					
28	2	0	2023	Zawiah, Mohammed					
29	2	0	2024	Hoffman, James M					
30	2	0	2024	Nesbit, Todd W					
31	2	0	2024	Schweitzer, Pamela					
32	2	0.01	2023	Abu heshmeh, Shrouq					
33	2	0	2025	Aungst, Timothy Dy					
34	2	0	2023	Gharaibeh, Lobna					
35	2	0	2024	Scott, Christopher M					
36	2	0	2025	Alsulami, Fahad T					
37	2	0	2025	Li, Sheng					
38	2	0	2022	Chok, Jay					
39	2	0	2024	Ashraf, Amir Reza					
40	2	0	2025	Miller, Victoria					
41	2	0	2022	Roosan, Don					
42	2	0	2024	Tichy, Eric					
43	2	0	2024	Fittler, Andras					
44	2	0	2023	Al-rawi, Mahmood Basil A					
45	2	0	2025	Chase, Aaron					
46	2	0	2024	Dipiro, Joseph T					
47	2	0	2025	Zavaleta-monestel, Esteban					
48	2	0	2024	Smoke, Steven					
49	2	0	2025	Most, Amoreena					

According to Price’s law, we identified nine high-producing authors in Chinese literature and forty nine in English literature (Table 1 provides detailed information). All these authors had collaborative groups in English literature (Figure 3a), however, only Yi Fang (“方翼” in Chinese), Xingang Li (“李新刚” in Chinese), Min Zhang (“张敏” in Chinese), Bikui Zhang (“张毕奎” in Chinese), Chong Zhang (“张翀” in Chinese) and Dong Liu (“刘东” in Chinese) had established their own research networks specifically within the domain of Chinese literature (Figure 3b).

### Institution

**Figure 4:**
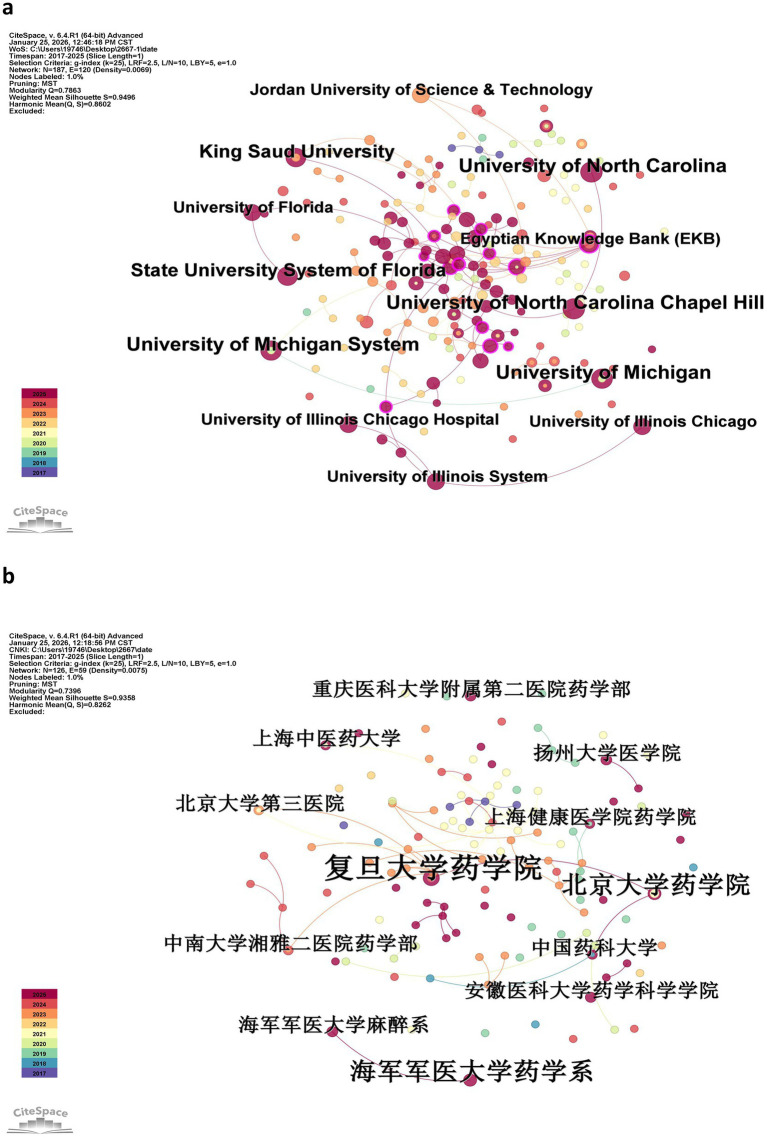
Map of cooperative relationship in English **(a)** and Chinese **(b)** literature publishing institutions in medical education related to CBL. In Chinese, the terms are organized as follows: “复旦大学药学院” for “Fudan University School of Pharmacy,” “北京大学药学院” for “Peking University School of Pharmacy,” “上海中医药大学” for “Shanghai University of Traditional Chinese Medicine,” “扬州大学医学院” for “Yangzhou University Medical College,” “中南大学湘雅二医院药学部” for “The Pharmacy Department of Xiangya Hospital of Central South University,” “中国药科大学” for “China Pharmaceutical University,” “安徽医科大学药学科学学院” for “School of Pharmacy, Anhui Medical University,”"海军军医大学麻醉系” for “Anesthesiology Department of Naval Medical University,” “海军军医大学药学系” for “Department of Pharmacy, Naval Medical University,” “北京大学第三医院” for “Peking University Third Hospital,” “上海健康医学院药学部” for “School of Pharmacy, Shanghai Health Science University,” “重庆医科大学附属第二医院药学部” for “The Pharmacy Department of the Second Hospital of Chongqing Medical University.” In the English literature network **(a)**, certain university systems and their specific campuses (e.g., “University of Michigan System” and “University of Michigan”) appear as distinct nodes. This reflects the raw hierarchical indexing formats inherent in the Web of Science database. While visualized separately to preserve the original metadata structure, they are analytically evaluated as consolidated entities in the text.

Figure 4 shows that there were 187 nodes and 273 connections in the English publishing institution, with a density of 0.0157 (Figure 4a), and 126 nodes and 141 connections with a density of 0.0179 in Chinese publishing institutions (Figure 4b). There are 8 institutions with the higher centrality, all more than or equal to 0.01, while other institutions in Chinese literature have a centrality of 0. In contrast, 9 institutions in the English literature exhibit a centrality exceeding 0.10, indicating a higher degree of overall inter-agency cooperation. The institutions with a large number of publications are shown in Table 2.

**Table 2:** Institutions with ≥4 papers published in English and Chinese.

English				Chinese			
No	count	Year	Institution	No	count	Year	Institution
1	8	2019–2025	University of Michigan (incl. University of Michigan System)	1	4	2021–2025	School of Pharmacy in Fudan University (复旦大学药学院)
2	8	2025	University of North Carolina (incl. UNC Chapel Hill)				
3	7	2023–2025	King Saud University				
4	7	2025	University of Florida (incl. State University System of Florida)				
5	5	2024–2025	Egyptian Knowledge Bank (EKB)				
6	5	2023–2025	Jordan University of Science and Technology				
7	5	2025	University of Illinois (incl. System and Chicago Hospital)				
8	4	2025	Harvard University				
9	4	2021–2025	Mayo Clinic				
10	4	2025	Pennsylvania Commonwealth System of Higher Education (PCSHE)				
11	4	2025	University of Georgia (incl. University System of Georgia)				
12	4	2020–2025	University System of Ohio				
13	4	2025	University of Colorado (incl. System and Anschutz Medical Campus)				

In English literature, following a comprehensive manual consolidation to resolve the hierarchical indexing of university systems, affiliated hospitals, and specific campuses (e.g., merging the distinct nodes of the University of Michigan, University of North Carolina, University of Florida, University of Illinois, University of Georgia, and University of Colorado into their respective primary institutions), the top-ranking institutions were the University of Michigan and the University of North Carolina, each associated with 8 articles. In Chinese literature, School of Pharmacy in Fudan University (“复旦大学药学院” in Chinese) ranked first with 4 papers.

### Keywords analysis

#### Keywords co-occurrence

**Figure 5:**
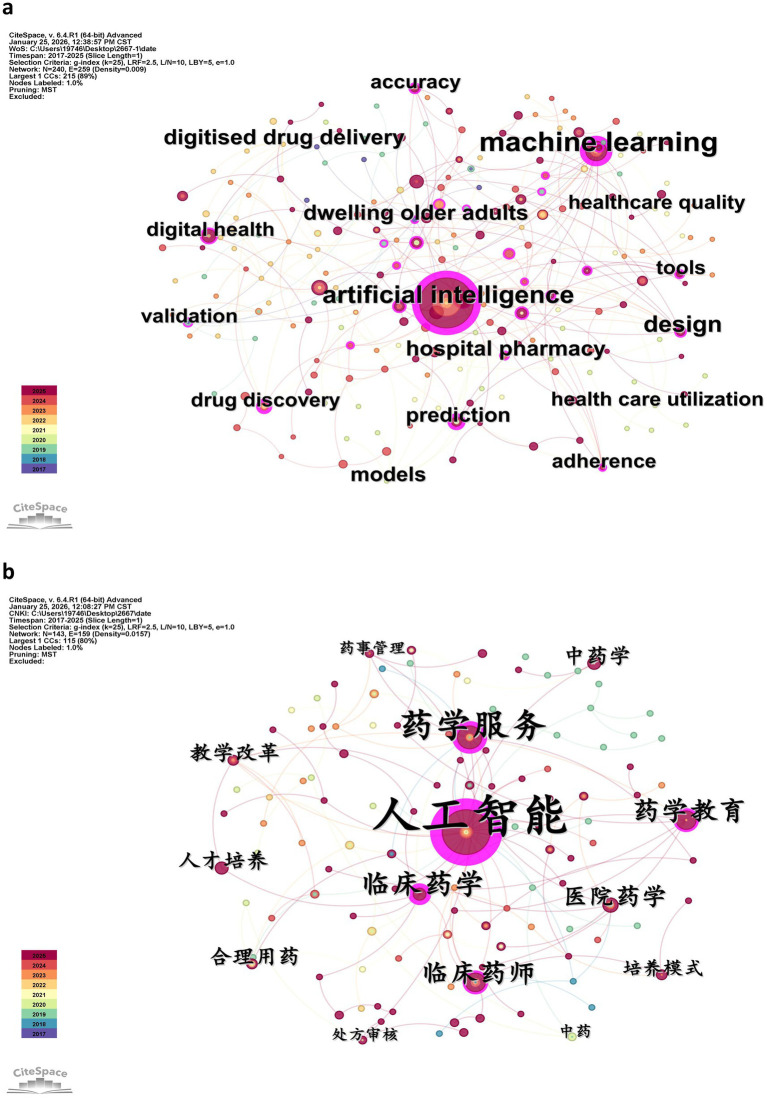
Knowledge map of keyword co-occurrence of English **(a)** and Chinese **(b)**. In Chinese, the terms are organized as follows: “人工智能” for “artificial Intelligence,” “药学服务” for “pharmaceutical care,” “药学教育” for “pharmacy education,” “临床药学” for “Clinical Pharmacy,” “临床药师” for “clinical Pharmacist,” “医院药学” for “hospital Pharmacy,” “药事管理” for “Pharmaceutical Management,” “中药学” for “traditional Chinese pharmacology,” “教学改革”for “teaching Reform,” “人才培养” for “personnel Training,” “合理用药” for “appropriate medication use,” “培养模式” for “training model,” “处方审核” for “prescription review,” “中药” for “traditional Chinese Medicine”.

Figure 5 displays the resulting keyword knowledge graph. Table 3 lists the top 10 keywords by frequency and centrality. The co-occurrence analysis and chart reveal distinct high-frequency and high-centrality keywords in both the Chinese and the English literature. Notably, the overlap between high-frequency and high-centrality keywords, such as “artificial intelligence,” is methodologically expected given their core thematic role in the respective datasets. Although these keywords are commonly used in other specific fields, their application varies between the Chinese and English literature. The English literature mainly concentrates on machine learning and pharmacy education, whereas the Chinese literature emphasizes pharmaceutical care and clinical pharmacists.

**Table 3:** High-frequency and high-centrality keywords in Chinese and English literature.

No	Dataset	High-frequency				High-centrality			
		Count	Centrality	Year	Keywords	Count	Centrality	Year	Keywords
1	Chinese	67	1.03	2018	artificial intelligence (人工智能)	67	1.03	2018	Artificial intelligence (人工智能)
2		20	0.13	2018	pharmaceutical care (药学服务)	20	0.13	2018	Pharmaceutical care (药学服务)
3		12	0.07	2020	clinical Pharmacy (临床药学)	3	0.09	2019	Traditional Chinese Medicine (中药)
4		9	0.08	2018	Clinical pharmacist (临床药师)	9	0.08	2018	Clinical pharmacist (临床药师)
5		9	0.06	2019	Pharmacy (药学)	12	0.07	2020	Clinical Pharmacy (临床药学)
6		9	0.03	2023	Pharmacy education (药学教育)	7	0.07	2018	Hospital Pharmacy (医院药学)
7		7	0.07	2018	Hospital Pharmacy (医院药学)	9	0.06	2019	Pharmacy (药学)
8		5	0.03	2020	Appropriate medication use (合理用药)	2	0.04	2019	Challenge (挑战)
9		5	0.02	2023	Teaching reform (教学改革)	2	0.04	2025	ERNIE Bot (文心一言)
10		5	0	2025	Personnel Training (人才培养)	5	0.03	2021	Traditional Chinese pharmacology (中药学)
1	English	128	0.73	2019	Artificial intelligence	128	0.73	2019	Artificial intelligence
2		28	0.38	2019	Machine learning	28	0.38	2019	Machine learning
3		12	0.12	2024	Pharmacy education	10	0.2	2024	Clinical pharmacy
4		10	0.2	2024	Clinical pharmacy	3	0.14	2017	Adverse drug events
5		9	0.09	2022	Deep learning	8	0.13	2019	Digital health
6		8	0.07	2024	Generative AI	12	0.12	2024	Pharmacy education
7		8	0.13	2019	Digital health	3	0.12	2019	Medication adherence
8		6	0.03	2020	Big data	5	0.11	2021	Drug discovery
9		6	0.02	2020	Health	3	0.11	2022	Adherence
10		6	0.06	2024	Pharmacy practice	9	0.09	2022	Deep learning

#### Keywords bursts

**Figure 6:**
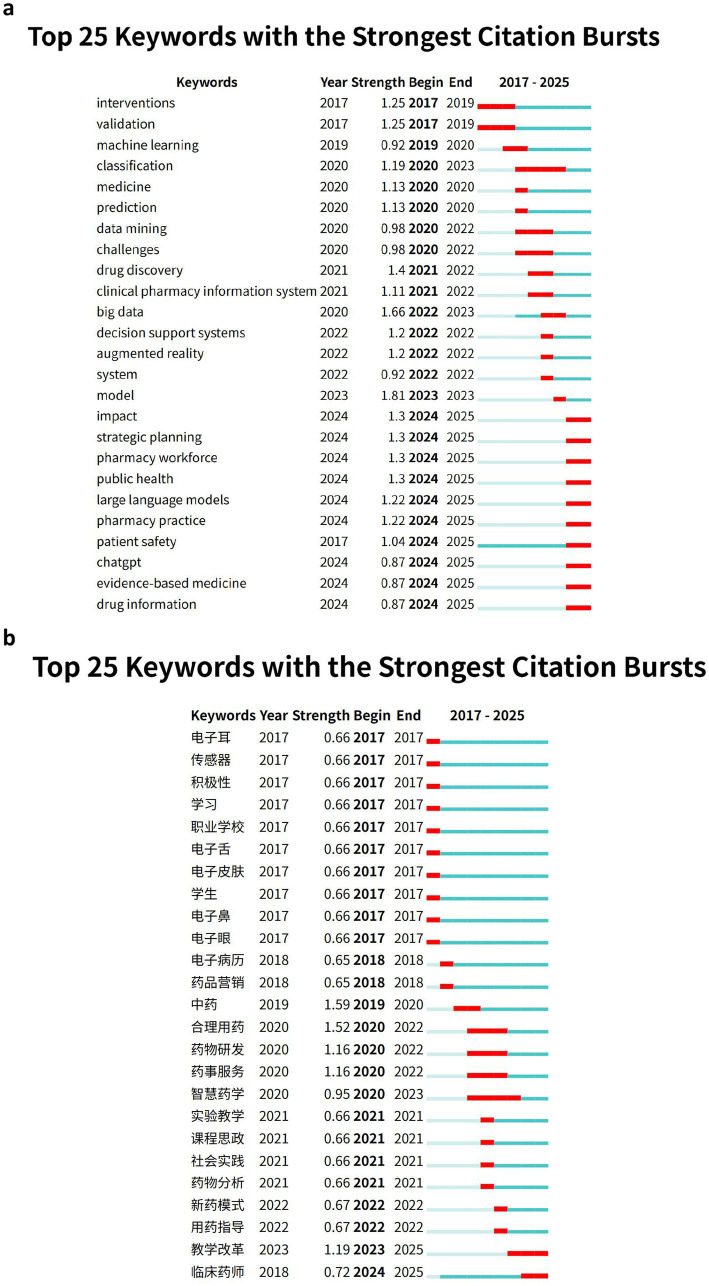
Top 25 keywords with strongest citation bursts in English **(a)** and Chinese **(b)** literature. In Chinese, the translations are as follows: “电子耳” for “electronic ear,” “传感器” for “sensor,” “临积极性” for “enthusiasm,” “学习” for “learning,” “职业学校” for “vocational School,” “电子舌” for “electronic tongue,” “电子皮肤” for “electronic skin,” “学生” for “students,” “电子鼻” for “Electronic nose,” “电子眼” for “electronic eye,” “电子病历” for “electronic medical record,” “药品营销” for “pharmaceutical marketing,” “中药” for “traditional Chinese Medicine,” “合理用药” for “rational drug use,” “药物研发” for “drug discovery,” “药学服务” for “pharmaceutical care,”"智慧药学” for “smart pharmacy,” “实验教学” for “experimental teaching,” “课程思政” for “curriculum ideology and politics,” “社会实践” for “social practice,” “药物分析” for “pharmaceutical analysis,” “新药模式” for “new drug model,” “用药组织” for “medicinal tissue,” “教学改革” for “teaching Reform,” and”临床药师” for “clinical pharmacist”.

As depicted in Figures 6a,b, the top 25 keywords in both the Chinese and the English literature were identified through keyword bursts analysis. Furthermore, based on the content found in both the English and the Chinese literature, these burst keywords can be categorized into distinct thematic groups, as presented in Table 4.

**Table 4:** The classification of keywords bursts and keywords cluster.

No	English		Chinese	
	Classification	Keywords burst	Classification	Keywords Burst
Keywords bursts				
1	Keywords related to AI and intelligent systems	Machine learning; big data; large language models; chatgpt; decision support systems; augmented reality; system	Keywords related to smart pharmacy and sensing technology	Smart pharmacy (智慧药学); electronic ear (电子耳); electronic tongue (电子舌); electronic nose (电子鼻); sensors (传感器); drug discovery (药物研发); new drug models (新药模式)
2	Keywords related to pharmacy practice and clinical systems	Clinical pharmacy information system; drug discovery; pharmacy practice; drug information; prediction; medicine	Keywords related to clinical pharmacy services and rational drug use	Rational drug use (合理用药); traditional Chinese medicine (中药); clinical pharmacists (临床药师); pharmaceutical analysis (药物分析); medication guidance (用药指导); electronic medical records (电子病历)
3	Keywords related to healthcare systems and safety	Interventions; validation; patient safety; public health; evidence-based medicine; impact	Keywords related to teaching reform and policy guidance	Teaching reform (教学改革); curriculum ideology and politics (课程思政); experimental teaching (实验教学); social practice (社会实践); enthusiasm (积极性)
4	Keywords related to strategic planning and models	Strategic planning; model; challenges; classification; prediction; validation	Keywords related to teaching evaluation and student development	Innovation ability (创新能力); teaching effectiveness (教学效果); quality education (素质教育); students (学生); vocational schools (职业学校)
Keywords cluster				
1	The research on care	#0 care*; #13 primary care	the research related to national policy guidance	#10 the ideological and political teaching (思政教学)
2	the research on community practice and special projects	#1 community of practice; #10 project echo; #11 teacher education	The research on teaching reform, mode and evaluation	#0 teaching methods (教学方法); #1 case teaching (案例教学); #2 teaching reform (教学改革); #4 scenario simulation (情景模拟)
3	The research on teaching methods	#2 blended learning; #4 evidence-based practice; #5 problem-based learning; #9 case-based teaching	The research related to various disciplines	#3 basic medicine (基础医学); #5 evidence-based medicine (循证医学); #6 nursing education (护理教育); #7 experimental teaching (实验教学)
4	The research on capacity development	#3 attitude; #6 clinical competence; #7 decision making; #8 clinical reasoning	The research related to clinical practice	#9 doctor-patient communication (医患沟通); #11 general family medicine (全科医学); #12 biochemistry (生物化学)

* The “Keywords bursts” section has been updated based on the 2017–2025 burst analysis from Figures 6a,b.

In the domain of English literature, the initial category includes keywords associated with AI and intelligent information processing. This category reflects a significant thematic interest in digital transformation. Key terms like “machine learning” (burst strength 0.92) appeared early in 2019, followed by “big data” (1.66) and “decision support systems” (1.2) in 2022. Most notably, “large language models” (1.22) and “ChatGPT” (0.87) emerged in 2024, representing the most recent frontier in pharmaceutical research and education.

The second category encompasses keywords related to pharmaceutical practice and clinical information systems. This group highlights the application of technology in clinical pharmacy. Keywords such as “drug discovery” (1.4) and “clinical pharmacy information system” (1.11) showed significant bursts around 2021. This trend is supplemented by recent bursts in “pharmacy practice” (1.22) and “drug information” (0.87) starting in 2024.

The third category of keywords pertains to healthcare systems and patient safety. A sustained emphasis on system-level improvements is evident. “Interventions” and “validation” both showed early bursts (1.25) in 2017. More recently, “patient safety” (1.04) and “public health” (1.3) have become prominent, suggesting a growing scholarly focus on broad health outcomes rather than just technical implementation.

The fourth category relates to methodological evolution and strategic planning. Terms such as “model” (1.81) and “impact” (1.3) highlight prominent methodological themes in pharmaceutical research. “Strategic planning” (1.3) and “challenges” (0.98) suggest that the field is currently navigating the complexities of integrating advanced technology into existing medical frameworks.

In Chinese literature, the first category of keywords is closely associated with smart pharmacy and sensing technology. This category reflects an early interest in biomimetic and sensory technologies. Between 2017 and 2018, a cluster of “electronic” keywords appeared, including “electronic ear” (电子耳), “electronic tongue” (电子舌), and “electronic nose” (电子鼻), all with a burst strength of 0.66. This laid the foundation for “smart pharmacy” (智慧药学, 0.95), which emerged as a burst term in 2020.

The second category of keywords pertains closely to clinical pharmacy services and rational drug use. This group underscores the practical application of pharmacy in clinical settings. “Traditional Chinese Medicine” (中药) showed a high intensity (1.59) starting in 2019, followed closely by “rational drug use” (合理用药, 1.52) and “clinical pharmacists” (临床药师, 0.72). These terms highlight the transition from basic research to patient-centered pharmaceutical care.

The third category pertains to teaching reform and policy guidance. This category aligns with national educational strategies and social responsibilities. Keywords such as “curriculum ideology and politics” (课程思政, 0.66) and “experimental teaching” (实验教学, 0.66) appeared in 2021. Most significantly, “teaching reform” (教学改革) shows a high burst intensity of 1.19 (2023–2025), indicating that systemic educational topics remain a core research focus.

The fourth category relates to teaching evaluation and student development. This group focuses on the outcomes of educational interventions. Keywords like “innovation ability” (创新能力), “teaching effectiveness” (教学效果), and “quality education” (素质教育) reflect an emphasis on the cultivation of comprehensive talents. The inclusion of “vocational schools” (职业学校) and “students” (学生) suggests that these educational improvements are being implemented across various levels of the academic hierarchy.

#### Keywords clustering and time diagram

**Figure 7:**
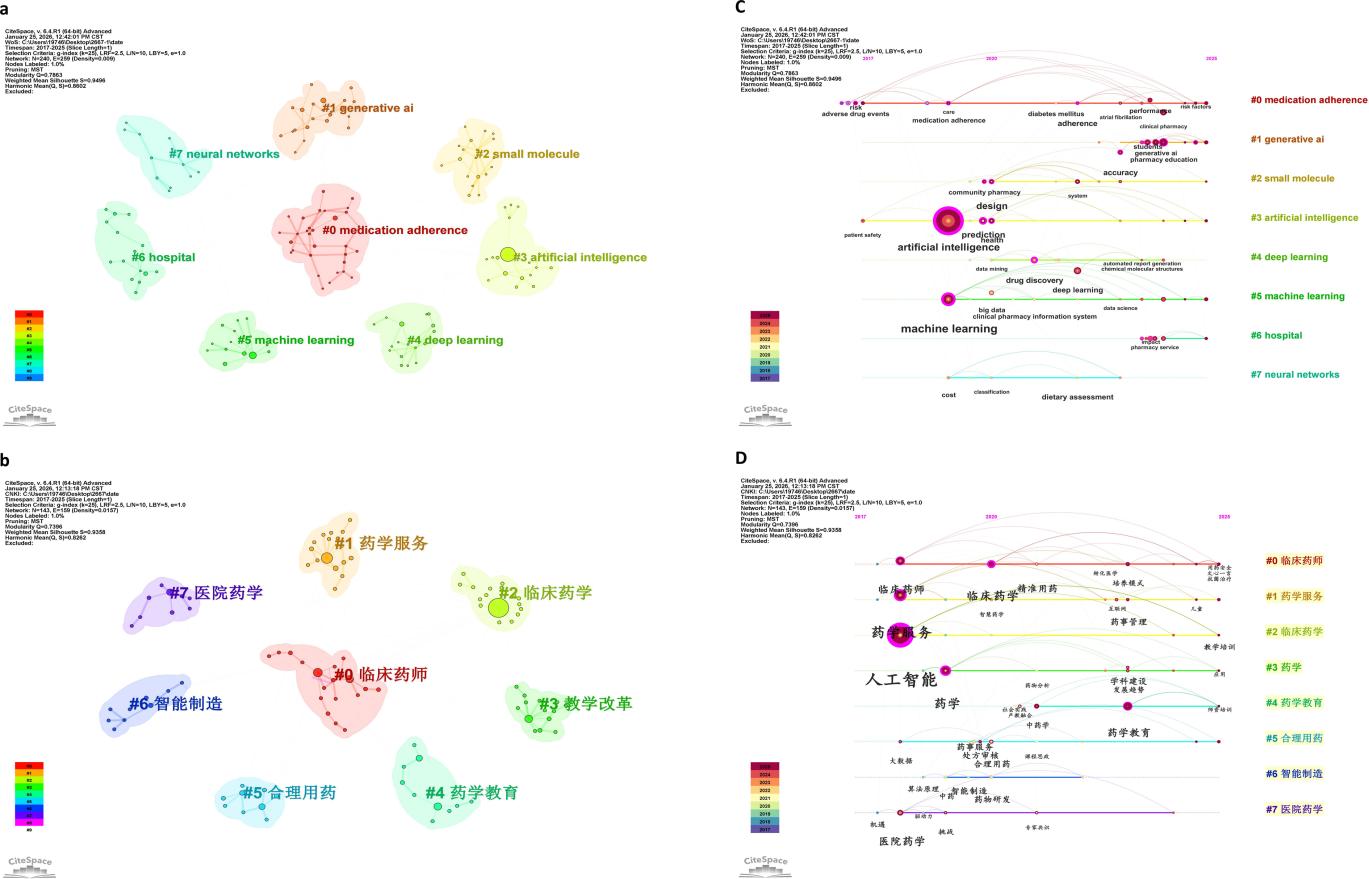
Keyword clustering diagram of **a** (English literature) and **b** (Chinese literature), while **c** (English literature) and **d** (Chinese literature) are the keyword clustering time diagram. Below the time line are the top 2 or 3 keywords that are cited most frequently in a particular year, and the most frequently cited keywords are lower. **b**: In Chinese, the cluster terms are as follows: “#0 临床药师” translates for “clinical pharmacists,” “#1 药学服务” for “pharmaceutical care,” “#2 临床药学” for “clinical pharmacy,” “#3 教学改革” for “teaching reform,” “#4 药学教育” for “pharmacy education,” “#5 合理用药” for “rational drug use,” “#6 智能制造” for “intelligent manufacturing,” and “#7 医院药学” for “hospital pharmacy.” **d**: In Chinese, the terms are organized as follows: First Row #0: “临床药师” for “clinical pharmacists,” “转化医学” for “translational medicine,” “培养模式” for “training model,” “用药安全” for “medication safety,” “文心一言” for “ERNIE Bot,” “抗菌治疗” for “antibacterial treatment.” Second Row #1: “药学服务” for “pharmaceutical care,” “互联网” for “Internet,” “儿童” for “children.” Third Row #2: “临床药学” for “clinical pharmacy,” “智慧药学” for “smart pharmacy,” “药事管理” for “pharmacy management,” “教学培训” for “teaching and training.” Fourth Row #3: “人工智能” for “artificial intelligence,” “应用” for “application.” Fifth Row #4: “药学” for “pharmacy,” “社会实践” for “social practice,” “产教融合” for “industry-education integration,” “学科建设” for “discipline construction,” “发展趋势” for “development trends,” “师资培训” for “teacher training.” Sixth Row #5: “合理用药” for “rational drug use,” “处方审核” for “prescription review,” “课程思政” for “curriculum ideology and politics.” Seventh Row #6: “大数据” for “big data,” “算法原理” for “algorithmic principles,” “智能制造” for “intelligent manufacturing,” “药物研发” for “drug R&D.” Eighth Row #7: “机遇” for “opportunities,” “挑战” for “challenges,” “驱动力” for “driving force,” “专家共识” for “expert consensus,” “医院药学” for “hospital pharmacy.

Figures 7a,b represent the cluster diagrams, while Figures 7c,d depict the time diagrams with red dots indicating keyword bursts. The clusters are coded starting from zero, where cluster #0 is the largest one that gradually decreases in size thereafter (Table 5).

**Table 5:** Top 8 clustering keywords in English and Chinese literature.

ID	Size	S	Mean (year)	Terms (mutual information)	ID	Size	S	Mean (year)	Terms (Chinese) (mutual information)
0	31	0.916	2020	Medication adherence (6.02, 0.05); explainable artificial intelligence (4.81, 0.05); antihypertensive therapy (4.81, 0.05); probability-based adherence stratification (4.81, 0.05); arrhythmia (4.81, 0.05)	0	19	0.946	2023	Clinical Pharmacist (临床药师) (11.78, 0.001); medical coordination management (医药协同管理) (8.13, 0.005); emergency treatment (急诊) (8.13, 0.005); community acquired pneumonia (社区获得性肺炎) (8.13, 0.005); antibacterial therapy(抗菌治疗) (8.13, 0.005)
1	23	0.875	2024	Generative AI (11.72, 0.001); pharmacy education (11.72, 0.001); pharmaceutical management (4.69, 0.05); chat-based ai tools (4.69, 0.05); procrastination (4.69, 0.05)	1	17	0.962	2021	Pharmaceutical care (药学服务) (14.03, 0.001); pharmacy management (药事管理) (7.99, 0.005); internet (互联网) (7.99, 0.005); smart pharmacy (智慧药学) (7.99, 0.005); practice (实践) (3.97, 0.05)
2	19	0.978	2021	Small molecule (10.96, 0.001); molecular property (5.48, 0.05); classification (5.48, 0.05); accuracy (2.62, 0.5); performance (2.62, 0.5)	2	17	1	2023	Clinical pharmacist (临床药师) (3.98, 0.05); clinical pharmacy (临床药学) (3.98, 0.05); artificial intelligence (人工智能) (3.5, 0.1); pharmacy education (药学教育) (3.17, 0.1); pharmaceutical care (药学服务) (2.35, 0.5)
3	18	0.944	2022	Artificial intelligence (4.53, 0.5); drug discovery (3.8, 0.5); natural language processing (1.91, 0.5); patient safety (0.9, 0.5); models (0.74, 1.0)	3	14	0.951	2021	Pharmacy (药学) (7.17, 0.01); drug clinical trials(药物临床试验) (4.9, 0.05); innovative applications (创新应用) (4.9, 0.05); application (应用) (4.9, 0.05); preclinical drug development (临床前药物研发) (4.9, 0.05)
4	17	0.928	2022	Deep learning (18.1, 1.0E−4); feature extraction (5.85, 0.05); models (4.23, 0.5); artificial intelligence (0.24, 1.0); prediction (0.1, 1.0)	4	10	0.853	2023	Pharmacy education (药学教育) (11.02, 0.001); integration of production and education (产教融合) (10.13, 0.005); cultivation system (培养体系) (5.01, 0.05); academic writing (学术写作) (5.01, 0.05); Top-notch innovative talents (拔尖创新人才) (5.01, 0.05)
5	15	0.994	2022	Machine learning (20.43, 1.0E−4); large language models (9.24, 0.005); clinical pharmacy information system (8.98, 0.005); big data (5.38, 0.05); medication use (4.47, 0.05)	5	9	0.824	2021	Rational drug use (合理用药) (11.53, 0.001); medication guidance(用药指导) (11.53, 0.001); prescription review (处方审核) (11.53, 0.001); prescription flow (处方流转) (5.68, 0.05); internet-based medical consultation(互联网诊疗) (5.68, 0.05)
6	15	1	2024	Hospital (16.76, 1.0E−4); pharmacy service (11.12, 0.001); healthcare technology (11.12, 0.001); patient care (7.43, 0.01); pharmacy practice (5.82, 0.05)	6	9	0.924	2019	Intelligent manufacturing (智能制造) (7.73, 0.01); expert system (专家系统) (7.73, 0.01); smart pharmaceuticals (智能制药) (7.73, 0.01); prescription design (处方设计) (7.73, 0.01); traditional Chinese medicine (中药) (7.73, 0.01)
7	13	0.941	2020	Neural networks (6.45, 0.05); medical decision making (6.45, 0.05); medication systems (5.43, 0.05); prediction (4.76, 0.05); machine learning (1.62, 0.5)	7	9	0.953	2019	Hospital pharmacy (医院药学) (8.16, 0.005); obtain employment (就业) (5.85, 0.05);high-level professional talents in traditional Chinese medicine (高职中药学人才) (5.85, 0.05); challenge (挑战) (5.85, 0.05); opportunities (机遇) (5.85, 0.05)

The network structure of English literature (WoS) is composed of 240 nodes and 259 edges, with a network density of 0.009. The Modularity Q value for this network is 0.7863, and the Weighted Mean Silhouette (S value) stands at 0.9496. In contrast, the Chinese literature (CNKI) network consists of 143 nodes and 159 edges, with a network density of 0.0157. The Q value is calculated as 0.7396, while the S value is 0.9358. According to CiteSpace evaluation criteria, a Q value greater than 0.3 indicates a significant clustering structure within the module, and an S value closer to 1 signifies higher homogeneity within the network. Generally, an S value exceeding 0.7 suggests that the clustering results are highly efficient and reliable. In this study, the Q and S values for both English and Chinese literature datasets far exceed these thresholds, demonstrating a highly robust clustering structure and credible analytical results.

Based on the visualization in Figures 7a,c, the 8 primary clusters in the English literature can be categorized into three major research domains:

- Artificial Intelligence and Computational Technologies (5 clusters): this is the most prominent research area, including #1 generative ai, #3 artificial intelligence, #4 deep learning, #5 machine learning, and #7 neural networks. The timeline indicates that these technologies are extensively applied to drug research, automated report generation, and the analysis of chemical molecular structures.
- Clinical Practice and Patient Care (2 clusters): this domain focuses on practical medical applications, specifically #0 medication adherence and #6 hospital. Research themes involve adverse drug event risks, clinical pharmacy service quality, and patient safety.
- Molecular Science and Drug Discovery (1 cluster): represented by cluster #2 small molecule, this area primarily explores systematic design and precision prediction within pharmaceutical development.

In contrast, the clustering content of Chinese literature can be classified into three categories:

- Pharmaceutical Care and Clinical Pharmacy Practice (5 clusters): this constitutes the core of Chinese research, comprising #0 Clinical Pharmacists (临床药师), #1 Pharmaceutical Care (药学服务), #2 Clinical Pharmacy (临床药学), #5 Rational Drug Use (合理用药), and #7 Hospital Pharmacy (医院药学). Key research focuses include translational medicine, prescription review, precision medication, and the exploration of pharmacy management models.
- Educational Reform and Talent Cultivation (2 clusters): this category emphasizes improvements in teaching, including #3 Teaching Reform (教学改革) and #4 Pharmacy Education (药学教育). Discussion centers on ideological and political teaching (Curriculum Ideology), industry-education integration, faculty training, and discipline construction under the “Internet+” era.
- Technological Application and Intelligent Manufacturing (1 cluster): represented by #6 Intelligent Manufacturing (智能制造). This field integrates big data and algorithmic principles into drug R&D, focusing on the intelligence of Traditional Chinese Medicine (TCM) and the enhancement of driving forces in pharmaceutical manufacturing processes.

## Discussion

### Publication trend

The application of AI in Pharmacy has mirrored the progression observed in other natural science domains, transitioning from a nascent exploratory phase to a period of steady, multifaceted growth. During the initial stage from 2017 to 2019, AI technology was in its early form, characterized by the foundational use of “electronic tongues” and “noses” for the quality control of Traditional Chinese Medicine (21). This soon evolved into a phase of steady growth (2020–2022), where the field moved beyond basic automation toward complex neural network implementations. Research during this period saw the robust application of Deep Neural Networks (DNNs) and Recurrent Neural Networks (RNNs) in drug discovery and pharmacokinetic prediction (22). Simultaneously, clinical settings began integrating AI into electronic patient records to manage polypharmacy (23) and deploying advanced hardware like the APOTECA chemo robot for precision compounding (24).

Since 2023, the field has entered a stage of rapid expansion, marked by a surge in large-scale data analysis and specialized intelligent services. Chinese literature, projected to peak in 2025 with 61 articles, has increasingly emphasized the “clinical problem-research-feedback” loop in translational medicine to cultivate high-level pharmaceutical talents (25), while exploring the synergy between AI and humanized services to redefine the pharmacist’s role (26). English literature, reaching its zenith in 2025 with 111 articles, has kept pace with contemporary global trends by investigating sophisticated themes such as explainable AI (XAI) for real-world health surveillance (27). This stage reflects a matured research environment where technical efficiency, ethical frameworks, and social acceptability are scrutinized in parallel to ensure the sustainable development of the smart pharmacy ecosystem (23, 27). It is noteworthy that the numerical imbalance in publication volume (240 English vs. 118 Chinese articles) should not be oversimplified as a mere difference in research productivity. This variance is fundamentally contextualized by structural factors: WoS encompasses a mature, global publication ecosystem with a broader multidisciplinary indexing scope, whereas CNKI captures a regionally focused, highly policy-driven academic environment. Thus, the comparison highlights distinct developmental trajectories and database inclusion criteria rather than purely quantitative output.

### Author co-occurrence analysis

The author serves as the fundamental driving force behind research, and identifying prolific authors and core groups provides an overview of the research field. Compared to other natural science domains, the number of published papers in the field of pharmaceutical AI is notably lower. This phenomenon can be attributed to the inherent complexity of integrating pharmacological mechanisms with computational algorithms; namely, the exploration and validation phase for target discovery and drug design tends to be relatively lengthy with most endeavors requiring extensive cross-disciplinary coordination (12). When a robust predictive model or a novel drug-target interaction is discovered, it is often refined and tested over several years to ensure clinical reliability rather than immediately pivoting to a different approach. This is in contrast to some branches of computer science where a single author might produce a high volume of output in a short span. In our study, the highest number of publications by a single author is merely 6 (Nelson, Scott D), and the field exhibits a publication centrality value of 0, indicating that no “burst” authors have emerged and research remains relatively fragmented. For example, Zhu Feng, a prominent figure identified in our analysis, led a massive systematic review in 2023 that encapsulated decades of advancements in AI-driven pharmaceutical sciences, and concurrently advocated for the “digital-intelligence pharmacy” framework to promote smart drug research innovation in 2022 (28). However, despite his central role in defining these academic structures, the output remains focused on high-quality, long-term systemic integration rather than rapid, high-frequency publishing. This suggests that the research in pharmaceutical AI is more focused on long-term, sustained efforts and the persistent convergence of digital technology and pharmaceutical science rather than rapid, high-volume output (12, 28).

### Research institution analysis

The institutions with the highest number of English and Chinese literature publications primarily consist of universities and their affiliated healthcare systems. Following a rigorous manual consolidation of hierarchically indexed branches, the University of Michigan (including the University of Michigan System) and the University of North Carolina (including UNC Chapel Hill) lead the English publications, with 8 papers each published consistently from 2019 to 2025. Their research strongly focuses on the integration of AI in pharmacy practice, medication systems, clinical decision-making, and pharmaceutical education. Following closely are King Saud University and the University of Florida (including the State University System of Florida), each having published 7 papers. Notably, over the past few years, the English literature has demonstrated a high degree of inter-agency cooperation, with institutions like the University of Michigan exhibiting high centrality values. The collaborative research networks across these global institutions effectively combine AI-driven predictive modeling with clinical outcomes such as medication adherence and patient safety.

The School of Pharmacy at Fudan University ranked first in the number of Chinese publications (4 papers) published annually from 2021 to 2025, primarily focusing on the application of AI in pharmaceutical care, clinical pharmacy practice, and the exploration of smart pharmacy management models. Other domestic publications, such as those from Peking University and China Pharmaceutical University, predominantly explore the implementation of AI across traditional Chinese pharmacology, intelligent manufacturing, and pharmacy education reform. Overall, it is evident that English literature research in this area is more advanced in terms of institutional collaboration and knowledge maturity than its Chinese counterparts, highlighting the need for increased inter-agency synergy and interdisciplinary integration within domestic research to foster continuous innovation in the intelligent pharmacy domain.

### Keywords analysis

#### Comparative analysis of global and Chinese orientations

The keyword co-occurrence and burst analyses reveal distinct regional orientations. English literature tends to prioritize algorithmic sophistication and data-driven clinical support, emphasizing keywords like “machine learning” and “neural networks” (29). This reflects a technology-driven paradigm aimed at enhancing predictive accuracy. Conversely, Chinese literature underscores the standardization of clinical pharmaceutical services and the reform of talent cultivation systems, highlighting terms such as “rational drug use” and “curriculum reform.” This highlights a policy-guided approach to systemic transformation, aligning closely with national initiatives like the “new quality productive forces” (新质生产力). Furthermore, the structural analysis reveals critical nodes with high betweenness centrality (e.g., “artificial intelligence” and “machine learning” in WoS; “pharmaceutical care” in CNKI). These high-centrality nodes act as essential transitional knowledge pathways. They indicate that AI is not an isolated technical subject but a bridging domain that connects foundational computational science with direct clinical practice and curricular design, thereby enriching the overarching educational framework.

#### Keywords bursts analysis

Our burst analysis reveals distinct evolutionary trajectories that directly impact how future pharmacy professionals must be trained. In the English literature, the strong burst of terms related to computational foundations, such as “drug discovery” and “machine learning” (Figure 6a), highlights a critical pedagogical shift. Rather than merely tracking technical developments, our bibliometric findings suggest that these domains are emerging as core competencies required in modern pharmacy training. For instance, while external studies frequently summarize the success of AI-driven models like AlphaFold in structural chemistry (30, 31), our burst analysis interprets this trend as a mandate for academic institutions to integrate computational modeling into foundational pharmacy curricula, moving away from traditional rote memorization.

Similarly, the prominent citation bursts for “clinical pharmacy information system” and “pharmacy practice” reflect a scholarly consensus that technology is rapidly transitioning into direct patient care. This bibliometric shift signifies that educators face the immediate challenge of training students in algorithm-assisted decision-making (32). As supported by external reviews evaluating AI-powered medication management tools in community settings, the focus of pharmacy education must pivot toward teaching students how to interpret and validate digital health interventions.

Furthermore, our burst analysis identified “challenges” and “strategic planning” as critical nodes representing professional readiness and implementation barriers. This bibliometric finding is corroborated by real-world empirical evidence; for example, regional surveys among healthcare students (such as those conducted in Riyadh) (33) and community pharmacists consistently reveal that despite a high awareness of AI’s potential, significant barriers remain, including a lack of technical infrastructure and formal training. Therefore, our data underscores the urgent need to bridge the gap between theoretical AI knowledge and practical clinical application through structured educational reform (34).

#### Keywords cluster analysis

The CiteSpace-based keyword clustering highlights a significant research frontier focused on the professional competence and educational readiness of pharmacy stakeholders as they navigate AI integration. Our network structure indicates a growing scholarly focus on the intersection of AI across various pharmaceutical domains, rather than supporting sweeping claims that AI has completely reshaped the entire sector (35, 36).

The prominence of Cluster #4 “deep learning” and Cluster #2 “small molecule” emphasizes the necessity of updating academic curricula. In the context of pharmacy education, the emergence of these clusters indicates that students must be trained to critically evaluate and utilize advanced models—such as AI-guided proteoformics for precision medicine or specific machine learning frameworks (e.g., the DELSTAR model) for predicting adverse drug events (37). These external examples serve as proof-of-concept for the bibliometric trends we observed, reinforcing that future pharmacists must transition from passive dispensers of medication to active managers of individualized, data-driven therapies (37–39).

Crucially, while many claims in the existing literature are framed as confirming AI’s benefits in optimizing patient care (40), interpreting our network results (such as the “patient safety” burst and “validation” node) necessitates a rigorous critical view. The bibliometric data indicates a pressing need for educational frameworks to address the inherent risks of these technologies. Despite repeated mentions of ethics, future curricula must aggressively incorporate training on identifying algorithmic bias in clinical decision support (41), managing AI “hallucinations” during patient counseling, navigating regulatory barriers, and ensuring the assessment validity of AI-generated medical knowledge in high-stakes environments.

To contextualize our clustering results regarding competency gaps and digital literacy, incorporating real-world workforce evidence is paramount. Recent studies examining pharmacy students’ and pharmacists’ knowledge, attitudes, and practices (KAP) toward artificial intelligence and precision medicine provide crucial support for our findings. For example, recent cross-sectional evaluations (42) highlight specific deficits in digital literacy and practical readiness among pharmacy students, while other empirical studies (43) demonstrate the urgent necessity of targeted professional readiness programs for AI adoption. These studies enrich our bibliometric observation that the transition to a “digital-intelligence pharmacy” requires not just technological acquisition, but a fundamental evolution in educational readiness and workforce preparedness.

## Conclusion

Based on the preceding discussion, we can draw the following conclusions regarding the bibliometric landscape of AI in the pharmacy sector:

- The body of literature regarding AI in pharmacy, much like other leading-edge natural sciences, exhibits a clear pattern of rapid, exponential-like growth. However, despite this rapid expansion, research remains primarily centralized within elite universities and their affiliated medical centers. Collaboration among diverse institutions and cross-industry partnerships is still relatively limited, with a significant portion of high-level research occurring within isolated academic units rather than through integrated global networks.
- Co-occurrence and keyword cluster analysis reveal that bibliometric evidence suggests increasing scholarly attention toward AI applications across the pharmaceutical value chain. This includes a growing body of literature discussing topics such as *de novo* drug discovery and proteoformics at the research stage, as well as the optimization of clinical decision support, Medication Therapy Management (MTM), and adverse drug reaction (ADR) prediction in direct patient care settings.
- The emergence of specific research bursts suggests that the thematic focus of AI in pharmacy literature evolves alongside broader technological and environmental shifts. Research focuses have transitioned from early predictive algorithms to the current integration of large language models (LLMs) and generative AI. These tools have been seamlessly incorporated into pharmacy services to meet the demands of the “Intelligence Era” and national digital health policies, particularly in the development of smart pharmacy frameworks and telepharmacy.
- In both clinical practice and educational environments, AI is effectively synergized with diverse methodologies to enhance professional outcomes. Whether integrated with immersive teaching models and virtual simulations in the pharmacy curriculum or combined with evidence-based clinical workflows such as therapeutic drug monitoring (TDM), AI acts as a multidisciplinary catalyst that is increasingly explored to cultivate the professional quality and critical thinking capabilities of pharmaceutical practitioners.

## Data Availability

The original contributions presented in the study are included in the article/supplementary material, further inquiries can be directed to the corresponding authors.
